# Climate model variability leads to uncertain predictions of the future abundance of stream macroinvertebrates

**DOI:** 10.1038/s41598-020-59107-y

**Published:** 2020-02-13

**Authors:** Karan Kakouei, Sami Domisch, Jens Kiesel, Jochem Kail, Sonja C. Jähnig

**Affiliations:** 1 Leibniz-Institute of Freshwater Ecology and Inland Fisheries (IGB), Department of Ecosystem Research, Berlin, Germany; 2 Freie Universität Berlin, Institute of Biology, Berlin, Germany; 3 Christian-Albrechts-University Kiel, Institute for Natural Resource Conservation, Department of Hydrology and Water Resources Management, Kiel, Germany; 4 University of Duisburg-Essen, Department of Aquatic Ecology, Essen, Germany

**Keywords:** Biodiversity, Freshwater ecology

## Abstract

Climate change has the potential to alter the flow regimes of rivers and consequently affect the taxonomic and functional diversity of freshwater organisms. We modeled future flow regimes for the 2050 and 2090 time horizons and tested how flow regimes impact the abundance of 150 macroinvertebrate species and their functional trait compositions in one lowland river catchment (Treene) and one mountainous river catchment (Kinzig) in Europe. We used all 16 global circulation models (GCMs) and regional climate models (RCMs) of the CORDEX dataset under the RCP 8.5 scenario to calculate future river flows. The high variability in relative change of flow among the 16 climate models cascaded into the ecological models and resulted in substantially different predicted abundance values for single species. This variability also cascades into any subsequent analysis of taxonomic or functional freshwater biodiversity. Our results showed that flow alteration effects are different depending on the catchment and the underlying species pool. Documenting such uncertainties provides a basis for the further assessment of potential climate-change impacts on freshwater taxa distributions.

## Introduction

The abundance of river biota and the resulting functional trait compositions of species communities are driven by environmental factors. Flow dynamics are known to regulate the species functional trait composition by determining the structure of the physical habitat and subsequent mechanisms, such as delivery of organic matter, in river ecosystems^1–4^. Climate change is projected to significantly alter natural flow regimes and dynamics^5–8^, thus affecting the composition and diversity of stream macroinvertebrates^2,9^. Concerns about the detrimental effects of climate change on river biota have increased in recent years^10–12^. Only recently have quantitative long-term observational flow data been used to model the quantitative flow preferences of stream macroinvertebrates^10,12^, which has been applied to predict potential changes in species abundance caused by flow alterations^13^. Such modeled quantitative preferences can be used to investigate the effects of flow alterations on the functional trait composition of river biota.

The response of species to climate change is frequently assessed using future projected environmental data and by modeling species distributions^14^. In streams and rivers, modeled flow alterations are often used to estimate potential climate change impacts on stream macroinvertebrates (e.g., the probability of occurrences or abundances)^11,12^, but little work has been done to assess the uncertainty in the projections of either flow alterations^7^ or the responses of river biota^14^. Instead, potential projected changes in the flow regime and their effects on river biota have been assessed according to either a single climate change scenario^12,13^ or by comparing the effects of various representative concentration pathways (RCPs)^7,15–17^. However, climate change predictions differ even within each RCP because different global circulation models (GCMs) are used, which are then downscaled to smaller domains using different regional climate models (RCMs) to provide high-resolution simulated data for regional or even smaller scales^6^. Since different GCMs and RCMs rely on a variety of model parameters, the future predicted climate for any given RCP can vary substantially depending on which GCMs and RCMs are selected. Consequently, the projected future flow alterations (from simulated discharge data) differ depending on the GCMs and RCMs used^18,19^. For example, Wang *et al*.^20^ reported the significant effects of a variety of downscaled GCMs on the frequency and sequence of flow events. Hence, the outcome of potential future biodiversity projections might also differ depending on the underlying GCMs and RCMs. An important task is therefore to assess the potential uncertainty regarding future biodiversity projections using all possible combinations of GCMs and RCMs.

Stream macroinvertebrates exhibit specific habitat requirements and flow preferences^21–23^; consequently, species can be categorized into trait-specific groups (e.g., rheophilic species^24^). The potential impacts of climate change on trait-based groups have been assessed more broadly by, for example, linking observed^22,23^ or modeled^25^ species range shifts and identifying cold-adapted species as the most vulnerable to warming climates given their range contractions. Traits related to flow alterations provide an additional detailed indicator of how climate change and the associated changes in flow regimes could impact species. To date, the effects of flow alterations on the functional trait composition have rarely been studied^12^, especially under the premise of relating functional traits to predicted abundances using a suite of different GCMs and RCMs.

The main goal of this study was to investigate the ecological effects of climate change on stream macroinvertebrates using all 16 available GCMs and RCMs of the CORDEX dataset^26^. More specifically, (i) we tested how this model variability cascades into the abundance models of stream macroinvertebrates and leads to uncertainties in our abundance predictions in a lowland catchment and a mountainous catchment in Central Europe. We hypothesized that (ii) species with narrow flow preferences would be strongly affected by climate change-induced flow alterations and that their predicted abundance would differ depending on the GCMs and RCMs, whereas generalist species tolerating a wide range of flows would be weakly affected by flow alterations and would not show differences between different GCMs and RCMs.

## Methods

### Study area and species data

**Figure 1:**
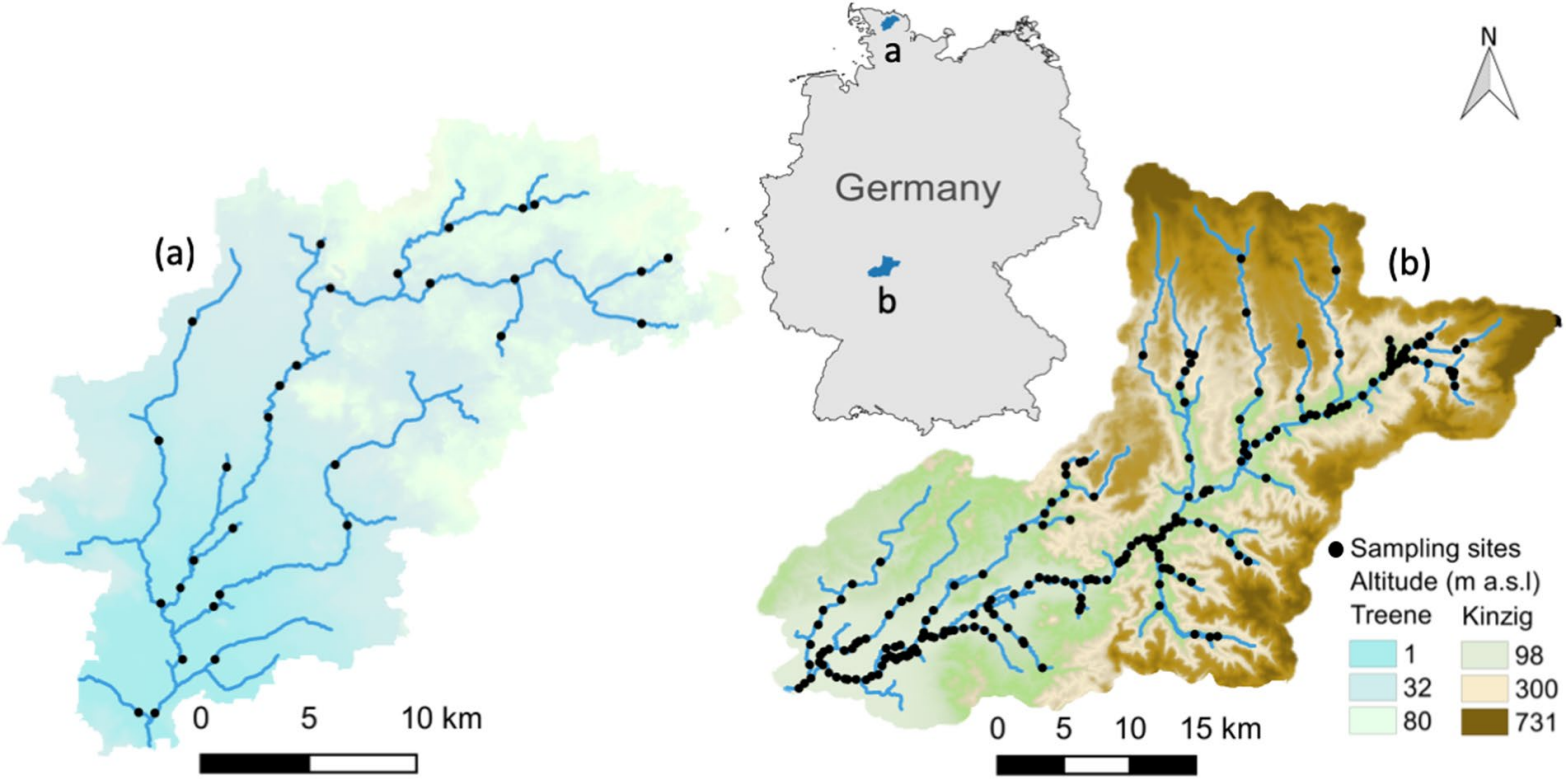
The study area^57^: The Treene catchment in the northern lowlands with 67 sampling sites (**a**) and the Kinzig catchment in the central lower mountainous region with 223 sampling sites (**b**) in Germany.

Our study areas were the Kinzig River catchment, characterized by a moderate gradient, fine-coarse sediment, surface runoff and interflow, and the Treene River catchment, with a small gradient, sand-gravel sediment, and groundwater-driven conditions, which are located in the central lower mountainous region and northern lowlands of Germany, respectively (Fig. 1)^13,27^. We used 134 macroinvertebrate species from 223 sampling sites in the central lower mountainous region and 60 species occurring at 67 sites in the northern lowlands, yielding 150 unique species from 15 taxonomic orders (Table ST1, see Kakouei *et al*.^13^ for the sampling procedure). Prior to modeling, we aimed to reduce potential uncertainties deriving from species data by excluding sampling sites with a “poor” or “bad” ecological status according to the monitoring required by the European Water Framework Directive.

### Modeling flow alterations resulting from different GCMs and RCMs

**Figure 2:**
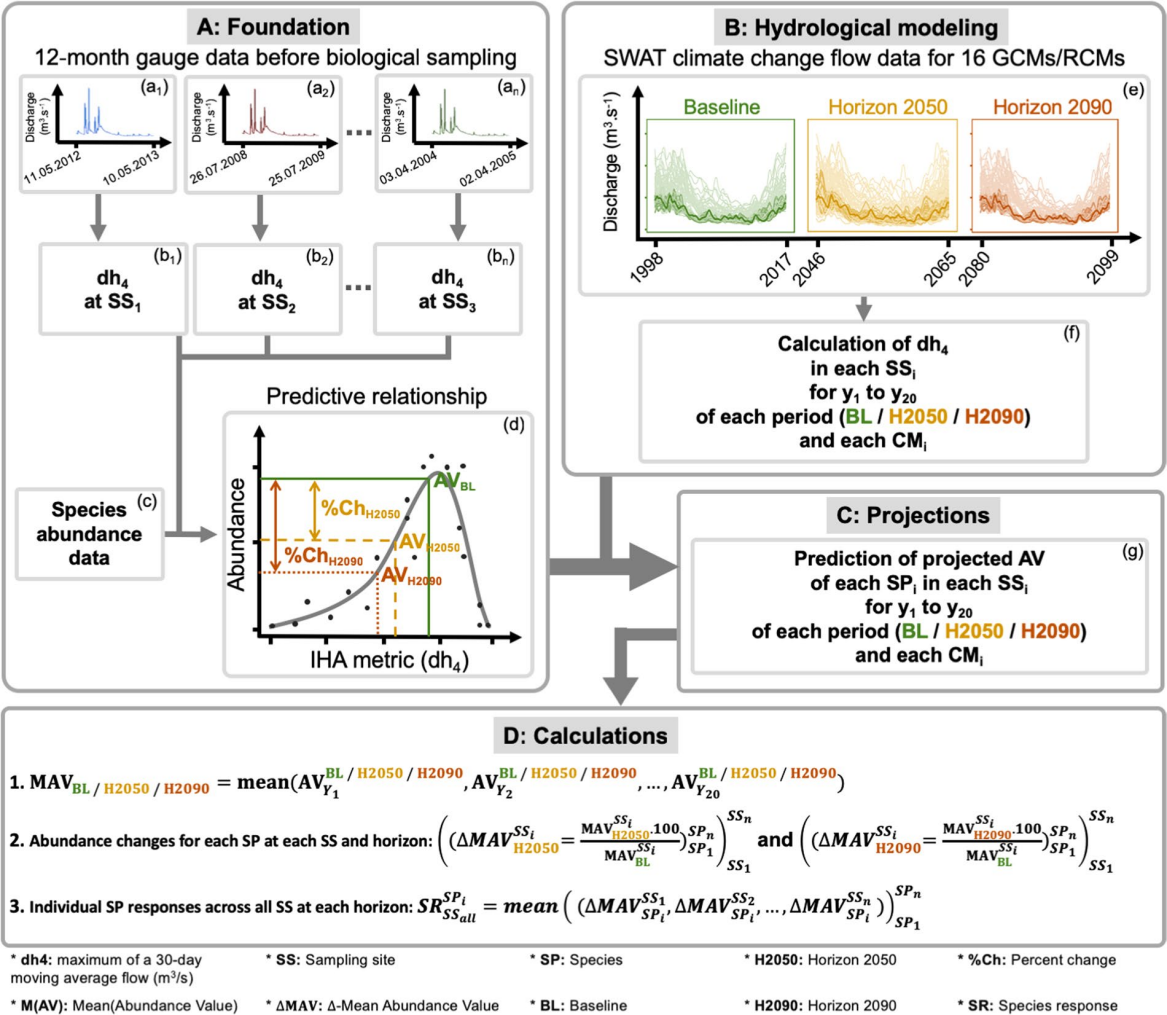
Workflow schematic of the analyses of both individual species and a single climate model^57^. We employed the predictive relationship (**A**) established by Kakouei *et al*.^13^ and employed only the dh4 IHA metric for each sample (b), using 12-month time-series gauge data collected before the date of biological sampling (a). Using the time-series discharge data that were provided for each of the 16 GCMs and RCMs (e), dh4 (f) was calculated for each year during the baseline (BL, e), horizon 2050 (H2050, e) and horizon 2090 (H2090, e) and then used to predict the projected abundance values (AV, g) for each species in each year during each period (g and d). The 20 abundance values per species were averaged to calculate the mean abundance value (MAV, D) for each species in each period. The projected changes in species abundance (SRs) were calculated by averaging the ΔMAV (**D**, equation 2) for each species among all the sampling sites (**D**, equation 3). All the analyses were repeated for each climate model (e,f) (Table ST2).

The projected daily flow data were modeled with the Soil and Water Assessment Tool (SWAT) ecohydrological model^28^. The models were forced with precipitation and minimum and maximum temperature datasets available from the CORDEX initiative at a daily resolution based on a 0.11° grid. All 16 currently available combinations of GCMs and RCMs for the study area (Table ST2) were downloaded from the CORDEX website^26^. In each case, we used RCP 8.5 as the future scenario, which represents the most extreme conditions and, hence, the upper limit for potential flow alterations and related taxon responses^13^. The CORDEX data were bias corrected (for precipitation: power transformation, and for temperature: variance scaling) and further downscaled to the SWAT subbasin resolution of approximately 40 km² on average. In the SWAT model application, we followed Kiesel *et al*.^29^ and used 12 hydrologically relevant parameters of the SWAT model, which were sampled 20,000 times each using Latin Hypercube sampling, as implemented by Pfannerstill *et al*.^30^. Then, a multiobjective calibration procedure^29^ was applied, from which the model parameterization that performed best in simultaneously depicting 32 indicators of hydrologic alteration (IHA) was selected. The function minimizes the Euclidean distance between the standardized IHA error residuals. A six-year period was used for calibration (2010–15) and a 12-year period for validation (1997–2009). The optimization of the model towards the multiobjective function of the 32 IHA yields very good agreement of the observed and simulated daily streamflow at the two gauges in the catchments. Model skill was assessed according to the Kling-Gupta-Efficiency (KGE^31^), which describes the agreement between the observed and simulated flow time series, and its value can range between negative infinity and 1, where a value of 1 denotes perfect agreement. The KGE is superior to the widely used Nash-Sutcliffe Efficiency (NSE) because when models are optimized towards the NSE, they tend to underestimate flow variability^32^. The model reached KGE values of 0.94 (NSE = 0.88) for the Treene and 0.93 (NSE = 0.87) for the calibration period in the Kinzig and achieved good agreement in the dh4 IHA (0.46 and 0.51 m³s^−1^ deviation in the Treene and Kinzig, respectively) used for the species simulations (Table 1). The bias-corrected, hindcast CORDEX data led to a better fit to the observations in the Treene than in the Kinzig (for more details, see Kiesel *et al*.^33^). The models were set up for three time periods: 1998–2017, 2046–2065, and 2080–2099, referred to as the baseline, horizon 2050, and horizon 2090, respectively (Fig. 2B). The impact of the only dam in the upstream region of the Kinzig catchment was considered in the hydrological model. The dam’s operation is based on release rules targeting individual reservoir water levels for the summer and winter periods, which were obtained from the reservoir operators.

**Table 1:** Detailed statistics for the daily streamflow simulations.

		Calibration (2010–2015)	Validation (1997–2009)
Treene	KGE	0.94	0.91
	NSE	0.88	0.84
Kinzig	KGE	0.93	0.87
	NSE	0.87	0.81

A wide range of flow metrics are available for describing the ecologically relevant characteristics of discharge time series, with the 171 indicators of hydrologic alteration (IHA)^34^ being the most widely used. Among the 171 IHA flow metrics, the “dh4” metric was selected, which describes the maximum 30-day moving average flow (m^3^ s^−1^) for the 12-month period prior to the date of biological sampling^34^. We selected this metric since it has been shown to have a significant impact on species abundance, yielding the highest predictive ability (excellent AUC values of 0.91 ± 0.03 and 0.94 ± 0.02 (mean ± standard deviation) in the Kinzig and Treene catchments, respectively) for macroinvertebrate species abundances in a previous study^13^. These predictive relationships were used in this study to predict the future abundance of individual species. Kakouei *et al*.^13^ used flow accumulation values (consecutive contributing areas) within a stream network to extrapolate time-series discharge data from subbasin outlets (provided by SWAT) to the sampling site of the representative subbasin. The extrapolated discharge time series were used to calculate the dh4 metric for each year in the three time periods at each sampling site.

### Potential effects of flow alterations on species abundance

Following Kakouei *et al*.^13^, the established predictive relationships for all species (Fig. 2A) were used in order to predict species abundances based on the dh4 flow metric. We calculated dh_4_ for each 12-month period within the three 20-year periods for each climate model (Fig. 2B). Using these predictive relationships (Fig. 2A) and the calculated dh_4_ values (Fig. 2B), we predicted the abundance of each individual species at each sampling site for each year of the three 20-year periods (for the baseline as well as 2050 and 2090, Fig. 2C) and then calculated the mean abundance values for each species at each sampling site in each period (Fig. 2D, equation 1). We aimed to further narrow the potential uncertainty stemming from the data itself by predicting the species abundances only at the sampling sites where the species were recorded, i.e., within the range predictions. Out-of-range predictions might be misleading due to missing environmental variables.

Given the modeled mean species abundances in the baseline time period, we calculated the decrease or increase in the mean abundance (delta value) for each individual species at each sampling site in the two horizon time periods, 2050 and 2090 (see Δ*MAV*, Fig. 2D, equation 2). These delta values were then summarized into the average percent change (%-Change, Fig. 2A) in the abundance of the individual species across all the sites where the species occurred (species responses [SRs], Fig. 2D, equation 3) for 2050 and 2090. This was repeated for all 16 GCMs and RCMs, resulting in 16 average delta values describing the projected abundance change (%-Change) for each species in 2050 and 2090.

In addition to these single climate model analyses, we also followed best practices in the field of climate studies^35^ and employed an ensemble of all 16 GCMs and RCMs using a weighted average. Here, the model performance of each RCM was judged according to the Euclidean distance between the IHA calculated from the simulated and baseline flow time series. The “simulated time series” were obtained from the hydrological models forced with the 16 hindcast CORDEX climate time series and the “baseline time series” from the simulations with the same hydrological model forced with the observed climate data. This ensured that the hydrological model error would not impact the assessment. A smaller Euclidean distance represents a better fit between simulations and observations and, hence, better model skill. We used a continuous weighting factor from one to two with intervals of 0.07 (i.e., 1 divided by the 16 GCMs and RCMs). The smaller the Euclidean distance is, the greater the weight of the respective GCMs and RCMs in the ensemble when assessing the relative change in species abundance according to that model. This resulted in a weighted average for each species, summarizing the projected abundance change across the 16 GCMs and RCMs.

### Assessing the effects of flow alterations on higher taxonomical levels and the functional trait composition

In addition to assessing the effects of flow alterations on a single species, we also grouped the species by taxonomic order and functional traits according to the *freshwaterecology.info* database^24^. We selected the following three flow-related functional traits for which information was available for at least 80% of the species in each catchment: (1) current preference, (2) stream zonation preference, and (3) feeding type (Table 2). Each trait (e.g., current preference) is described by several categories (e.g., a preference for a fast- or slow-flowing current). Each category is based on a 10-point assignment scale, where “10” describes a very strong affinity of a given species for a trait and “0” describes no affinity (*freshwaterecology.info*^24^).

We used an affinity value of ≥7 as an indicator of a strong preference of a given species for a trait category. In contrast, if the affinity values were spread across categories, we assumed that the species was a generalist. Regarding feeding types, the preferences for two trait categories (e.g., grazer-shredder) were selected only if a species had at least five points assigned in each category (for details, see Table 2).

All the statistical analyses were carried out in R 3.3.2^36^. For significance tests, we used one-way analysis of variance (ANOVA). Paired *t*-tests were used for the analyses between study sites. Percent data were normalized using an arc-sine transformation prior to the analyses^37^.

**Table 2:** Functional traits of stream macroinvertebrates (freshwaterecology.info-database^24^).

Traits	Categories
Current preference	Limnobiont Limnophil
	Limno- to rheophil
	Rheo- to limnophil Rheophil
	Rheobiont
	Indifferent
	Unknown
Stream zonation preference	Upstream
	Mid- to upstream
	Mid- to downstream
	Indifferent
	Unknown
Feeding type	Predator Grazer Shredder Gatherer Active filter feeder Passive filter feeder Grazer-shredder Grazer-gatherer Gatherer-shredder Generalist

## Results

### Flow alterations resulting from different GCMs and RCMs

**Figure 3:**
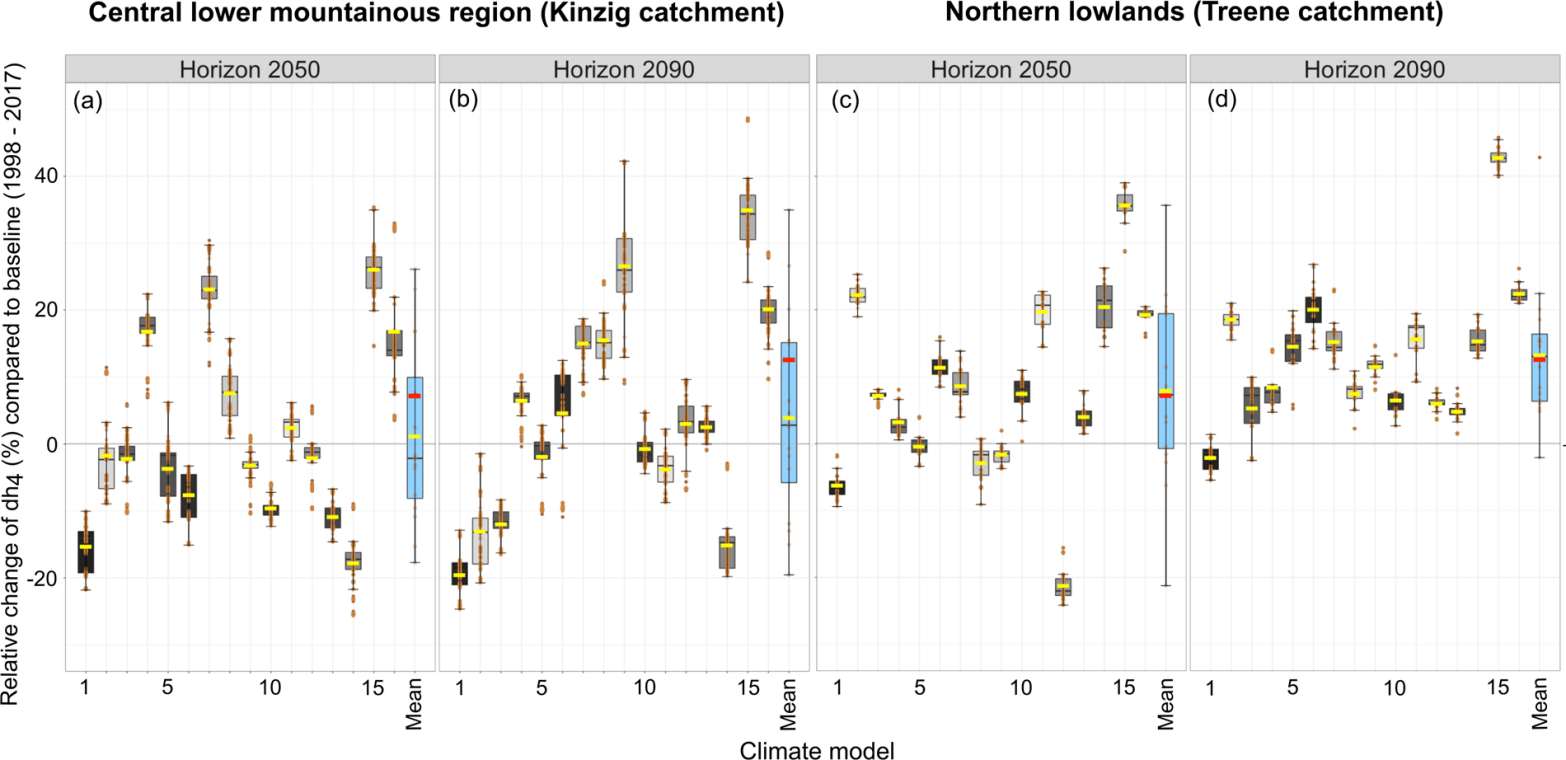
The boxplots (black bar – median; yellow bar - mean; red bar – weighted mean; box – 1^st^ and 3^rd^ interquartile ranges) show the projected relative change in flow conditions across all sampling sites in the Kinzig (n = 223) catchment in the central lower mountainous region (**a**,**b**) and the Treene (n = 67) catchment in the northern lowlands (**c**,**d**) for the two defined 20-year periods of horizon 2050 (2046–2065, **a** and **c**) and horizon 2090 (2080–2099, **b**,**d**) compared to the baseline (1998–2017)^57^. The blue boxplots show the variability across all hydrological models of the respective catchment and horizon. Each blue boxplot consists of 16 values representing the mean flow (dh4) changes across all sampling sites of each catchment and horizon compared to the baseline. The yellow horizontal lines are the means, and the red lines in the blue boxplots are the weighted means (for detailed information on the coefficient of variation belonging to each boxplot, see Table ST3).

Overall, the individual GCMs and RCMs predicted a substantial increase or decrease compared to the zero-change line in the flow conditions in both lowland and mountainous catchments (Figs. 2, 3a–d), as described by the dh4 metric, which is an important flow metric describing 30-day maximum flows^13^.

When pooling the results across sampling sites over all 16 GCMs and RCMs, a substantial change was predicted in the lowland catchment but not in the lower mountain catchment (Fig. 3). The weighted mean of the relative changes in the 30-day maximum flows (Fig. 3, red horizontal lines in blue boxplots) showed a substantial positive mean change in the northern lowlands in both horizons (+ 7.9% horizon 2050, +13.3% horizon 2090). In the central lower mountainous region, the weighted means were slightly different from zero.

### Predicting the effect of flow alterations on species abundance

**Figure 4:**
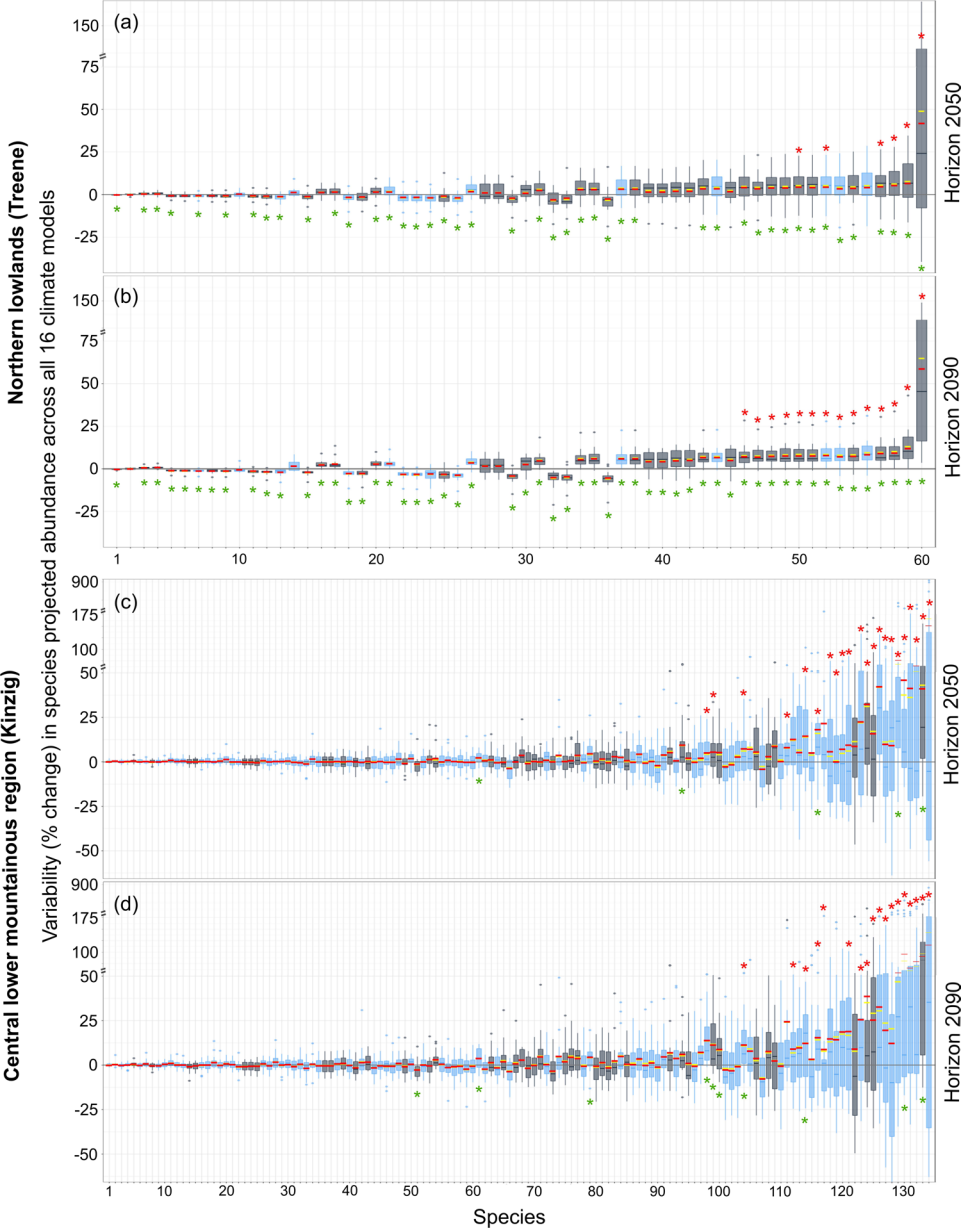
The variability in the projected abundance of individual species (SRs) in the central lower mountainous region (**a**,**b**) and the northern lowlands (**c**,**d**) across all 16 GCMs and RCMs (i.e., 16 values per species box plot) in horizon 2050 (**a**,**c**) and horizon 2090 (**b**,**d**). A significantly (*t*-test, p < 0.05) large overall weighted mean of the relative change in the abundance of each species compared to the average value across all species and all climate models is indicated by red asterisks () above the boxplots. A significantly (*t*-test, p < 0.01) different overall mean value from zero is indicated by green asterisks () below the boxplots. Gray boxplots represent species that occur in both catchments (for detailed information on species names and coefficients of variation belonging to each boxplot, see Figs. SF1 and SF2 and Tables ST4 and ST5).

The high variability in the relative change in dh4 between the 16 climate models cascaded into the ecological models resulted in 16 substantially different predicted abundance values for single species (Fig. 4). Following the relationship between the magnitude of the alterations in the 30-day maximum flows (Fig. 3) and the magnitude of the abundance change, the uncertainty in the predicted changes in species abundance was larger in the central lower mountainous region than in the northern lowlands (Fig. 4, Tables ST3, ST4). Furthermore, the abundance of 44 species that occur in both catchments (gray boxplots in Fig. 4, SF1 and SF2) was predicted to be more uncertain in the central lower mountainous region compared to the northern lowlands.

The relative variability (%-change) in species abundance across all species showed substantially higher uncertainties in the Kinzig compared to the Treene catchment. The key piece of evidence that supported this result was the greater number of species in the Kinzig that showed a significantly larger weighted mean across all 16 climate models compared to the absolute mean variability in species abundances across all species and all climate models. The absolute mean values of the overall changes in species abundance for horizons 2050 and 2090 were 2.8% and 5.1% in the Treene and 6.1% and 7.8% in the Kinzig, respectively. The relative changes in the abundance of only six and 14 species (horizons 2050 and 2090) in the Treene were significantly above the absolute mean; however, 18 and 22 species (horizons 2050 and 2090) showed substantially larger values in the Kinzig catchment (*t*-test, p < 0.05, red asterisks above the boxplots in Fig. 4a–d). The weighted means of the changes in the abundance of individual species over all 16 GCMs and RCMs showed that the abundances of 4% (n = 5) and 8% (n = 10) of species would significantly increase in horizons 2050 and 2090, respectively, in the central lower mountainous region (Fig. 4a,b). The changes were insignificant for the remaining species in this catchment. In the northern lowlands, the abundance was predicted to increase significantly for 73% (n = 44) of species in horizon 2050 and for 92% (n = 55) of species in horizon 2090 (green asterisks in Fig. 4a–d, *t*-test, p < 0.5).

The generalists (i.e., the 5% of species on the left side of Fig. 4) were substantially less sensitive (t-test, P < 0.01) than the specialists (i.e., the 5% of species on the right side of Fig. 4) to flow alterations.

### Assessing the effect of flow alterations on the functional trait composition

**Figure 5:**
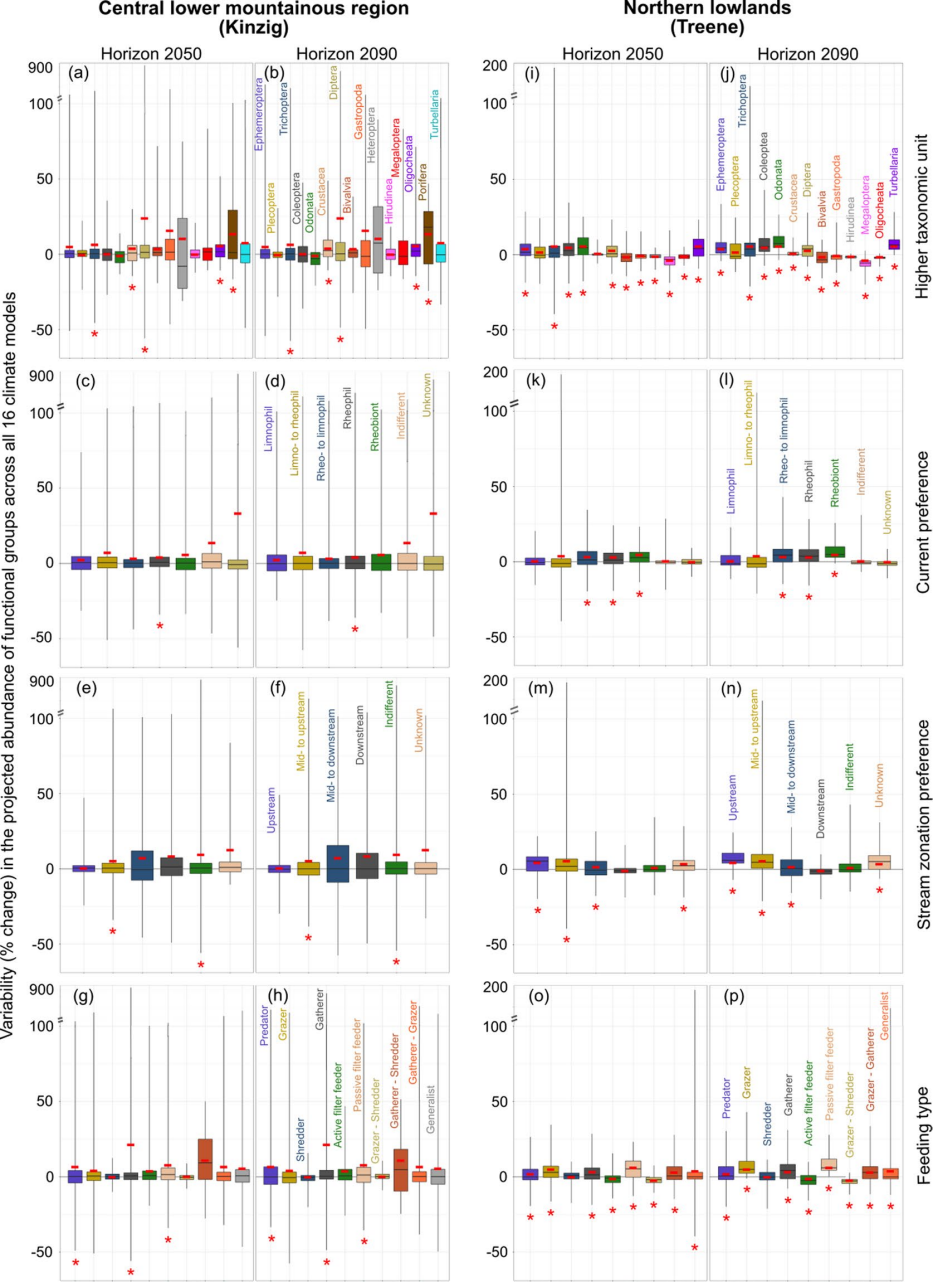
Variability in the projected abundance of the higher taxonomic units (**a**,**b**,**i**, and **j**) and functional groups (all others) in the central lower mountainous region (left column) and the northern lowlands (right column) to flow alterations across all 16 GCMs and RCMs and all sampling sites for horizon 2050 and horizon 2090. The red asterisks () indicate whether the overall weighted mean value (red hyphen in the middle of the boxplots) of the relative change in abundance among all the species in each functional group across all 16 GCMs and RCMs is significantly different from zero.

The community structure in terms of higher taxonomic units was predicted to change in both catchments. Among the widespread Ephemeroptera, Plecoptera and Trichoptera (EPT taxa) of the central lower mountainous region, significant changes across all 16 GCMs and RCMs were predicted only for Trichopera species (Fig. 5a,b). In the northern lowlands, the weighted mean abundance across all 16 GCMs and RCMs was predicted to increase significantly for Ephemeroptera and Trichoptera by 2–3% (*t*-test, p < 0.05, Fig. 5i,j). Considering the entire range of variability, including the whiskers, Ephemeroptera and Trichoptera in the central lower mountainous region and Trichoptera in the northern lowlands were predicted to exhibit the highest variability in projected abundance across the 16 GCMs and RCMs (−40 to 194% and −19 to 34%, respectively).

Species with rheophilic and mid- to upstream preferences were predicted to significantly increase in the northern lowlands, while significant increases were predicted for species with rheophilic and mid- to upstream preferences (both comprising one functional group only, see Fig. 5) in the central lower mountainous region (paired *t*-test, p < 0.5, Fig. 5c–f,k–n).

All the functional feeding groups except for shredders in the northern lowlands showed significant changes (−6 to 59% compared to the baseline) according to the weighted mean abundance (paired *t*-test, p < 0.5, Fig. 5o,p).

## Discussion

Our set of 16 possible combinations of GCMs and RCMs confirmed the outcome of previous studies that used a suite of GCMs^20,35,38,39^ and showed strong variation in the projected flow conditions. This high variability in turn cascaded into the ecological models and led to high variability in the predicted abundances of the individual stream macroinvertebrate species and their associated functional trait compositions.

The effects of flow alterations on microhabitats and, subsequently, species preferences and abundances differed between the two study areas most probably due to differences in climatic patterns and hydrological regimes (as suggested by Jourdan *et al*.^40^, and Lawrence *et al*.^41^; Pyne and Poff^12^; Theodoropoulos *et al*.^42^). The projected streamflow magnitudes significantly increase in the RCP 8.5 climate scenario in the northern lowland catchment, whereas the change is predicted to be insignificant in the lower mountainous region. Similar to our results, the duration and frequency of high-flow events in the northern lowlands has been reported to increase to a greater extent than that in the central lower mountainous region^43,44^, where the overall mean of annual streamflow shows decreasing trends^45^.

The variety of species responses to flow alterations addressed in different studies^11^ might be caused by the variety of the GCMs or RCMs or the scenarios used in each study. Ecological studies assessing the effects of climatic change on biological diversity often use single climate models or (non)weighted multimodel ensembles from either the RCP 4.5 or RCP 8.5 climate scenario. In such studies, the uncertainty in the projected response of species is usually defined as the observed range between the predictions according to single or multimodel ensembles of RCP 4.5 or 8.5. Multimodel ensembles have the potential to provide more accurate simulations and results compared to those of single models^39,46,47^ by increasing the skill, reliability and consistency of the projections^35,38,39,46,48^. However, the cascading effect of high variability across climate models on species abundance may be overlooked, as the weighted means are single values with high uncertainties. In our study, however, this high variability was not overlooked, as all the GCMs and RCMs were considered. This variability is observable in the high uncertainty in species projected abundances in the central lower mountainous region, which reflects the high variability in the projections of the 16 different GCMs and RCMs. This variability stems from the GCMs and RCMs, highlighting the uncertainty inherent in climate projections^35^. Another reason for the high uncertainty in species projected abundances in the central lower mountainous region might be the inferior climate model fit; i.e., the models were assessed according to the goodness of fit between the observations and the hindcast simulations for the historical period. This possibility is in agreement with Kotlarski *et al*.^49^, who demonstrated a decreasing model fit in this region compared to that in the northern lowlands for the CORDEX climate change data.

The metric describing the high-flow conditions in this study is inherently co-correlated with 68 of 151 IHA metrics from the same or even other IHA categories, thus representative for a variety of flow conditions described by these IHA metrics. Although more research in similar regions is needed to strengthen the outcomes, our results suggest that in regions where the variability across GCMs and RCMs is large and bracketed around the zero-change line, flow alterations are likely to be rendered insignificant. In such regions, the projection of species responses based on multimodel ensembles might then be misleading or at least challenging to interpret and might only be used to visualize trends. This shortcoming can be alleviated by improving the climate model skill, e.g., through advanced computational capacities supported by accurate long-term observation data. This approach can provide a basis for substantially reducing climate model uncertainties^35^, which may result in more reliable and robust predictions.

Similar to Kakouei *et al*.^50^, we reduced the uncertainty in the ecological models and abundance projections by (1) excluding sampling sites with a “poor” or “bad” ecological status according to the European Water Framework Directive and (2) validating the high predictive ability of the ecological models using a cross-validation approach, indicating the values of the area under the ROC curve (AUC, for details, see Kakouei *et al*.^50^). In addition, (3) we further reduced potential uncertainties by restricting the predictions to the sampling sites where the species were recorded. This prevented the prediction of possible false positives at locations where the species may not occur, thus decreasing the uncertainty in the predictions in the absence of other environmental variables or abiotic factors. Despite this conservative assumption, under which species are not predicted to migrate to new locations, the uncertainties became apparent when focusing only on the locations where the species were sampled. Nevertheless, the high uncertainty in the projected species abundances stemming from climate model variability makes focusing on the ecological meaning of these changes difficult. This is especially applicable to the lower mountainous region. Predicting species abundance based on the weighted means (red horizontal lines in the boxplots of Figs. 3–5) of discharge projections following the recommended multimodel ensemble method^35,38^ aids in visualizing the trends once changes in species abundance are predicted to be significant.

Our results suggest that the abundance of a variety of functional traits and, hence, community structure will be strongly affected by flow alterations. For example, the significant increasing trend in river flows in the northern lowlands is predicted to affect the species inhabiting this region, resulting in significantly increased or decreased abundances depending on the species and the low variability of their responses. This is in line with the patterns in the responses of functional groups, such as the significantly increasing abundance of rheophilic and tolerant rhithral species with upstream preferences in the northern lowlands. This suggests potential range extensions of these species, which is referred to as the “rhithralisation effect”^51,52^.

Species showed stronger responses at sampling sites with greater flow alterations but also with higher uncertainties. Generalists (i.e., the 5% of species with a wide distribution range on the left side of Fig. 4) are predicted to show less vulnerability to flow alterations, while the abundance of specialists (i.e., the 5% of species with a narrow distribution range on the right side of Fig. 4) that have narrow niches and occur at few sampling sites in the upstream region, for example, is predicted to respond strongly to changes in flow conditions. For example, generalists with wide ranges of current preferences, such as *Lype reducta* (according to Schmidt-Kloiber and Hering^24^ and Kakouei *et al*.^10^), are less likely to experience decreasing abundance resulting from reduced streamflows^9,53^. Our results for the Treene catchment showed that the abundance of these species (i.e., mainly species with indifferent current preferences, Fig. 5k,l) is predicted to change slightly, with small variability across the 16 GCMs and RCMs. Our findings might help ecologists to select target species in assessing species distributions across large spatial scales, which provide the necessary information that enable conservationists to choose the optimal protected areas to conserve specialist species against climate change and anthropogenic impacts.

Due to our focus on the changes in species abundance in the whole catchment, we ignored site-specific species losses by averaging the percent changes in the abundance of individual species across the sampling sites at which the species was present. Nevertheless, site-specific decreases in the abundance of species that play particular functional roles in communities may affect the functioning of river ecosystems. For instance, even the small decrease in the abundance of shredders (grazer-shredders in Fig. 5o,p) that is predicted to occur in the Treene catchment may affect functioning of these river ecosystems by influencing nutrient transfer and aquatic food webs^54,55^.

### Outlook

Our results showed uncertainties in the projected abundance of stream macroinvertebrates stemming from the variability among GCMs and RCMs. While it is known that different GCMs and RCMs lead to differences in streamflow predictions^33^ and their selection are a major source of uncertainty in climate change impact studies^18^, the magnitude by which they influence abundance of biota has been assessed for the first time here. We can conclude that these impacts are not negligible and hence, documenting such uncertainties is vital and provides a basis for the further assessment of potential climate change impacts on freshwater taxon distributions. Despite these uncertainties in hydrological models and their cascading effects on the ecological models, the range of changes in species’ abundances was substantially larger in the lower mountainous region, which has higher slopes and faster runoff response, as compared to the lowlands. The results of this study highlight the importance of maintaining natural flow conditions for riverine biota, providing of which might be a challenging water management issue, especially in the lower mountainous region. Furthermore, the taxa that showed high uncertainties and strong responses to flow alterations might be interesting to be used in a change index in climate-change impact assessments.

Although other environmental variables such as temperature changes also affect stream macroinvertebrates, our results show that river flows are already strongly influencing future species abundances and occurrences. Therefore, considering river flows and their alterations induced by climatic change in future research may complement research that addresses the effects of multiple stressors on river biota. The evaluation of species flow preferences provides a promising avenue for assessing the possible effects of flow alterations on stream macroinvertebrates. The methods used in this study should be applied to any flow conditions that particularly describe frequently occurring extreme events such as drought, described by IHA metrics, or any environmental variable describing various categories, such as climate (e.g., temperature) or water quality (e.g., pesticides, pollutants, or pH), to assess the effects of global changes on river ecosystems at different spatial scales. This may require spatially and temporally high-quality data and is possible by isolating species responses along a gradient of single stressors and removing sites where species are affected by a second stressor, as described in Hering *et al*.^56^. Given the upward trajectory of global warming, it is mandatory to understand the quantitative responses of stream macroinvertebrates to changes in environmental conditions.

## Data availability

All data related to the projected relative changes in flow conditions as well as species’ abundances and functional groups generated for this study are available at the following repository link: https://zenodo.org/record/3630325#.XjF55chKhaR.

## Supplementary information

Supplementary information.
