# Supplementary material for: Climate model variability leads to uncertain predictions of the future abundance of stream macroinvertebrates
Source: Sci Rep. 2020 Feb 13;10:2520. doi: 10.1038/s41598-020-59107-y (PMC7018820; doi:10.1038/s41598-020-59107-y)
Supplement: Supplementary file 1 — Supplementary information. [file 41598_2020_59107_MOESM1_ESM.docx]

# Title

Climate model variability leads to uncertain predictions of the future abundance of stream macroinvertebrates

# List of authors

Karan Kakouei^1,2,^*, Sami Domisch^1^, Jens Kiesel^1,3^, Jochem Kail^4^ and Sonja C. Jähnig^1^

*^1^ Leibniz-Institute of Freshwater Ecology and Inland Fisheries (IGB), Department of Ecosystem Research, Berlin, Germany*

*^2^ Freie Universität Berlin, Institute of Biology, Berlin, Germany*

*^3^ Christian-Albrechts-University Kiel, Institute for Natural Resource Conservation, Department of Hydrology and Water Resources Management, Kiel, Germany*

*^4^ University of Duisburg-Essen, Department of Aquatic Ecology, Essen, Germany*

* Corresponding author: Karan Kakouei, kakouei@igb-berlin.de

# Supplementary Information

## Figures


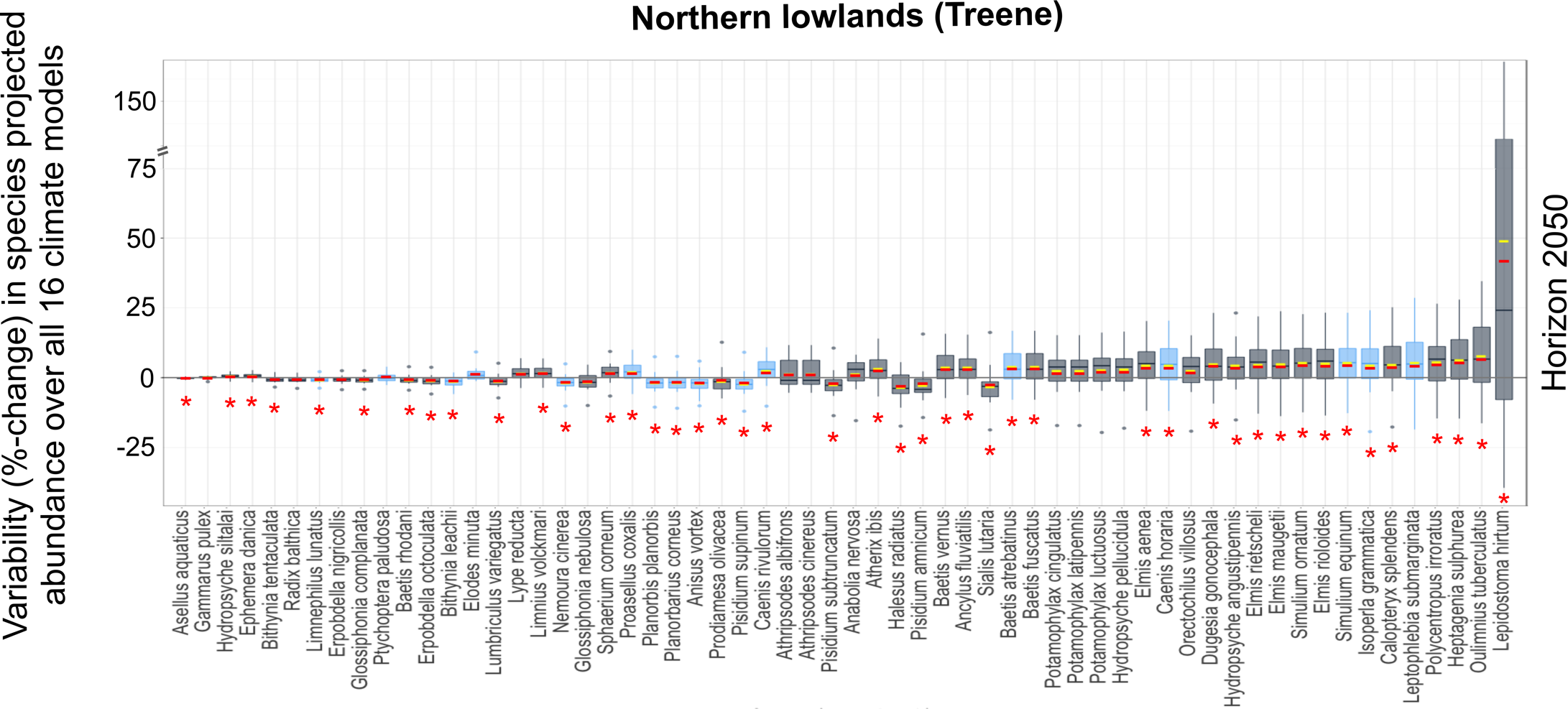


**Fig. SF1** Species name and the projected variability in their abundance in the northern lowlands over all 16 GCMs and RCMs (i.e. 16 values per species’ box-plot) in horizon 2050 and horizon 2090.


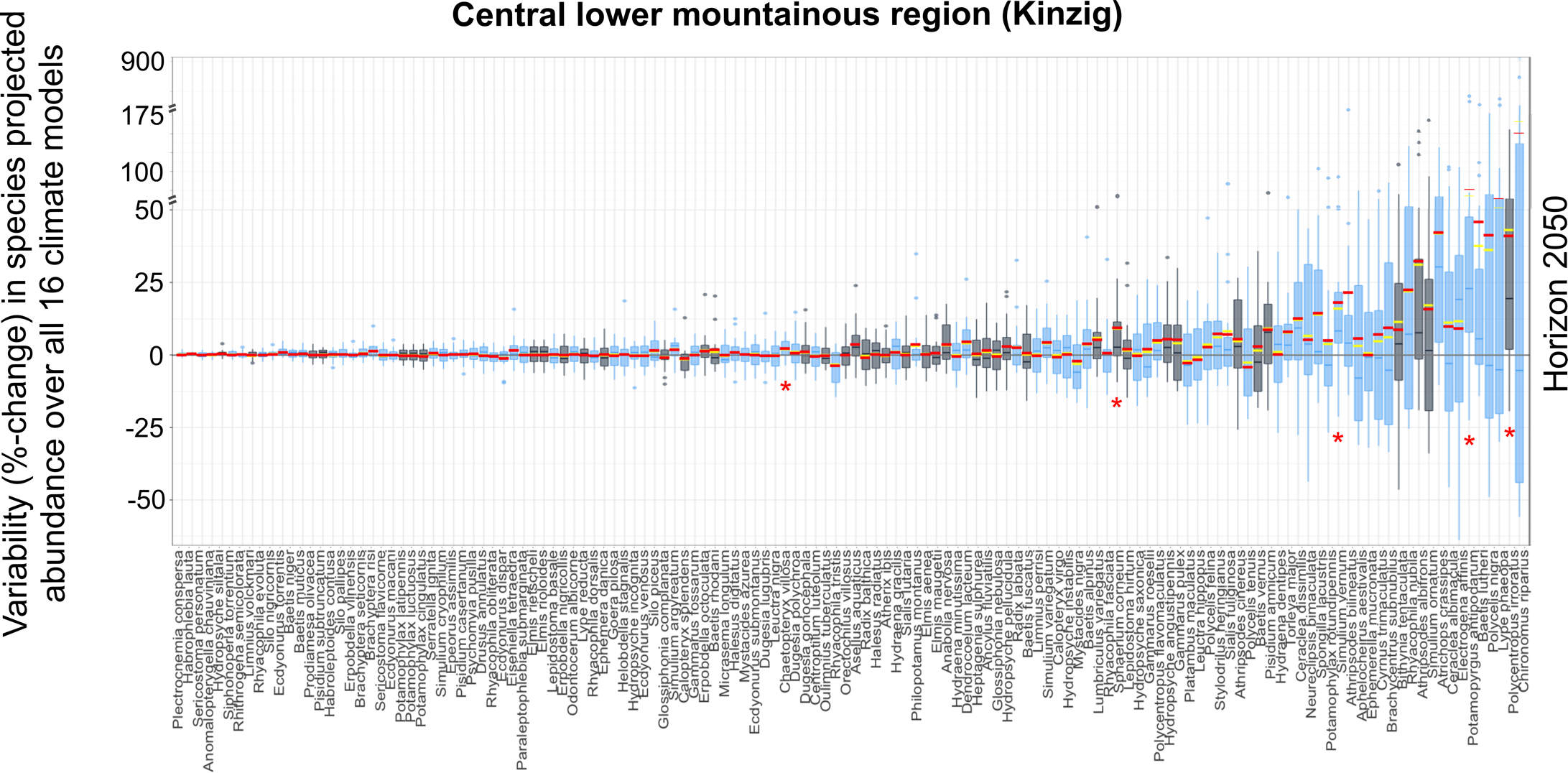


**Fig. SF2** Species name and the projected variability in their abundance in the central lower mountainous region (Kinzig catchment) over all 16 GCMs and RCMs (i.e. 16 values per species’ box-plot) in horizon 2050 and horizon 2090.

# Tables

**Table ST1** List of all 134 and 60 species of stream macroinvertebrates in the central lower mountainous region (Kinzig catchment) and northern lowlands (Treene catchment), respectively, and the author and higher taxonomical unit.

| **Species** | **Author** | **Higher taxa** | **Treene** | **Kinzig** |
| --- | --- | --- | --- | --- |
| *Pisidium amnicum* | O.F. MÜLLER, 1774 | Bivalvia | X | X |
| *Pisidium casertanum* | POLI, 1791 | Bivalvia | - | X |
| *Pisidium subtruncatum* | MALM, 1855 | Bivalvia | X | X |
| *Pisidium supinum* | A. SCHMIDT, 1851 | Bivalvia | X | - |
| *Sphaerium corneum* | (LINNAEUS, 1758 | Bivalvia | X | X |
| *Elmis aenea* | MÜLLER, 1806 | Coleoptera | X | X |
| *Elmis maugetii* | LATREILLE, 1798 | Coleoptera | X | X |
| *Elmis rietscheli* | STEFFAN, 1958 | Coleoptera | X | X |
| *Elmis rioloides* | KUWERT, 1890 | Coleoptera | X | X |
| *Elodes minuta* | LINNAEUS, 1767 | Coleoptera | X | - |
| *Hydraena dentipes* | GERMAR, 1844 | Coleoptera | - | X |
| *Hydraena gracilis* | GERMAR, 1824 | Coleoptera | - | X |
| *Hydraena minutissima* | STEPHENS, 1829 | Coleoptera | - | X |
| *Limnius perrisi* | DUFOUR, 1843 | Coleoptera | - | X |
| *Limnius volckmari* | PANZER, 1793 | Coleoptera | X | X |
| *Orectochilus villosus* | MÜLLER, 1776 | Coleoptera | X | X |
| *Oulimnius tuberculatus* | MÜLLER, 1806 | Coleoptera | X | X |
| *Platambus maculatus* | LINNAEUS, 1758 | Coleoptera | - | X |
| *Asellus aquaticus* | LINNAEUS, 1758 | Crustacea | X | X |
| *Gammarus fossarum* | KOCH in PANZER, 1836 | Crustacea | - | X |
| *Gammarus pulex* | LINNAEUS, 1758 | Crustacea | X | X |
| *Gammarus roeselii* | GERVAIS, 1835 | Crustacea | - | X |
| *Proasellus coxalis* | DOLLFUS, 1892 | Crustacea | X | - |
| *Atherix ibis* | FABRICIUS, 1798 | Diptera | X | X |
| *Atrichops crassipes* | MEIGEN, 1820 | Diptera | - | X |
| *Chironomus riparius* | MEIGEN, 1804 | Diptera | - | X |
| *Prodiamesa olivacea* | MEIGEN, 1818 | Diptera | X | X |
| *Ptychoptera paludosa* | MEIGEN, 1804 | Diptera | X | - |
| *Simulium argyreatum* | MEIGEN, 1838 | Diptera | - | X |
| *Simulium cryophilum* | RUBZOV, 1959 | Diptera | - | X |
| *Simulium equinum* | LINNAEUS, 1758 | Diptera | X | - |
| *Simulium ornatum* | MEIGEN, 1818 | Diptera | X | X |
| *Simulium variegatum* | MEIGEN, 1818 | Diptera | - | X |
| *Simulium vernum* | MACQUART, 1826 | Diptera | - | X |
| *Baetis alpinus* | PICTET, 1843-1845 | Ephemeroptera | - | X |
| *Baetis atrebatinus* | LEACH, 1815 | Ephemeroptera | X | - |
| *Baetis fuscatus* | LINNAEUS, 1761 | Ephemeroptera | X | X |
| *Baetis lutheri* | MÜLLER-LIEBENAU, 1967 | Ephemeroptera | - | X |
| *Baetis muticus* | LINNAEUS, 1758 | Ephemeroptera | - | X |
| *Baetis niger* | LINNAEUS, 1761 | Ephemeroptera | - | X |
| *Baetis rhodani* | PICTET, 1843-1845 | Ephemeroptera | X | X |
| *Baetis vernus* | CURTIS, 1834 | Ephemeroptera | X | X |
| *Caenis horaria* | LINNAEUS, 1758 | Ephemeroptera | X | - |
| *Caenis rivulorum* | EATON, 1884 | Ephemeroptera | X | - |
| *Centroptilum luteolum* | MÜLLER, 1776 | Ephemeroptera | - | X |
| *Ecdyonurus dispar* | CURTIS, 1834 | Ephemeroptera | - | X |
| *Ecdyonurus macani* | THOMAS & SOWA, 1970 | Ephemeroptera | - | X |
| *Ecdyonurus submontanus* | LANDA, 1969 | Ephemeroptera | - | X |
| *Ecdyonurus torrentis* | KIMMINS, 1942 | Ephemeroptera | - | X |
| *Ecdyonurus venosus* | FABRICIUS, 1775 | Ephemeroptera | - | X |
| *Electrogena affinis* | EATON, 1886 | Ephemeroptera | - | X |
| *Epeorus assimilis* | EATON, 1885 | Ephemeroptera | - | X |
| *Ephemera danica* | MÜLLER, 1764 | Ephemeroptera | X | X |
| *Ephemerella mucronata* | BENGTSSON, 1909 | Ephemeroptera | - | X |
| *Habroleptoides confusa* | SARTORI & JACOB, 1986 | Ephemeroptera | - | X |
| *Habrophlebia lauta* | EATON, 1884 | Ephemeroptera | - | X |
| *Heptagenia sulphurea* | MÜLLER, 1776 | Ephemeroptera | X | X |
| *Leptophlebia submarginata* | LINNAEUS, 1767 | Ephemeroptera | X | - |
| *Paraleptophlebia submarginata* | STEPHENS, 1835 | Ephemeroptera | - | X |
| *Rhithrogena semicolorata* | CURTIS, 1834 | Ephemeroptera | - | X |
| *Serratella ignita* | PODA, 1761 | Ephemeroptera | - | X |
| *Torleya major* | KLAPÁLEK, 1905 | Ephemeroptera | - | X |
| *Ancylus fluviatilis* | O.F. MÜLLER, 1774 | Gastropoda | X | X |
| *Anisus vortex* | LINNAEUS, 1758 | Gastropoda | X | - |
| *Bithynia leachii* | SHEPPARD, 1823 | Gastropoda | X | - |
| *Bithynia tentaculata* | LINNAEUS, 1758 | Gastropoda | X | X |
| *Planorbarius corneus* | LINNAEUS, 1758 | Gastropoda | X | - |
| *Planorbis planorbis* | LINNAEUS, 1758 | Gastropoda | X | - |
| *Potamopyrgus antipodarum* | GRAY, 1843 | Gastropoda | - | X |
| *Radix balthica* | LINNAEUS, 1758 | Gastropoda | X | X |
| *Radix labiata* | ROSSMÄSSLER, 1835 | Gastropoda | - | X |
| *Aphelocheirus aestivalis* | FABRICIUS, 1794 | Heteroptera | - | X |
| *Erpobdella nigricollis* | BRANDES, 1900 | Hirudinea | X | X |
| *Erpobdella octoculata* | LINNAEUS, 1758 | Hirudinea | X | X |
| *Erpobdella vilnensis* | LISKIEWICZ, 1925 | Hirudinea | - | X |
| *Glossiphonia complanata* | LINNAEUS, 1758 | Hirudinea | X | X |
| *Glossiphonia nebulosa* | KALBE, 1964 | Hirudinea | X | X |
| *Helobdella stagnalis* | LINNAEUS, 1758 | Hirudinea | - | X |
| *Sialis fuliginosa* | PICTET, 1836 | Megaloptera | - | X |
| *Sialis lutaria* | LINNAEUS, 1758 | Megaloptera | X | X |
| *Calopteryx splendens* | HARRIS, 1782 | Odonata | X | X |
| *Calopteryx virgo* | LINNAEUS, 1758 | Odonata | - | X |
| *Eiseniella tetraedra* | SAVIGNY, 1826 | Oligochaeta | - | X |
| *Lumbriculus variegatus* | MÜLLER, 1774 | Oligochaeta | X | X |
| *Stylodrilus heringianus* | CLAPAREDE, 1862 | Oligochaeta | - | X |
| *Brachyptera risi* | MORTON, 1896 | Plecoptera | - | X |
| *Brachyptera seticornis* | KLAPALEK, 1902 | Plecoptera | - | X |
| *Isoperla grammatica* | PODA, 1761 | Plecoptera | X | - |
| *Leuctra hippopus* | KEMPNY, 1899 | Plecoptera | - | X |
| *Leuctra nigra* | OLIVIER, 1811 | Plecoptera | - | X |
| *Nemoura cinerea* | RETZIUS, 1783 | Plecoptera | X | - |
| *Siphonoperla torrentium* | PICTET, 1841 | Plecoptera | - | X |
| *Spongilla lacustris* | LINNAEUS, 1758 | Porifera | - | X |
| *Anabolia nervosa* | CURTIS, 1834 | Trichoptera | X | X |
| *Anomalopterygella chauviniana* | STEIN, 1874 | Trichoptera | - | X |
| *Athripsodes albifrons* | LINNAEUS, 1758 | Trichoptera | X | X |
| *Athripsodes bilineatus* | LINNAEUS, 1758 | Trichoptera | - | X |
| *Athripsodes cinereus* | CURTIS, 1834 | Trichoptera | X | X |
| *Brachycentrus subnubilus* | CURTIS, 1834 | Trichoptera | - | X |
| *Ceraclea albimacula* | RAMBUR, 1877 | Trichoptera | - | X |
| *Ceraclea dissimilis* | STEPHENS, 1836 | Trichoptera | - | X |
| *Chaetopteryx villosa* | FABRICIUS, 1789 | Trichoptera | - | X |
| *Cyrnus trimaculatus* | CURTIS, 1834 | Trichoptera | - | X |
| *Drusus annulatus* | STEPHENS, 1837 | Trichoptera | - | X |
| *Goera pilosa* | FABRICIUS, 1775 | Trichoptera | - | X |
| *Halesus digitatus* | SCHRANK, 1781 | Trichoptera | - | X |
| *Halesus radiatus* | CURTIS, 1834 | Trichoptera | X | X |
| *Hydropsyche angustipennis* | CURTIS, 1834 | Trichoptera | X | X |
| *Hydropsyche incognita* | PITSCH, 1993 | Trichoptera | - | X |
| *Hydropsyche instabilis* | CURTIS, 1834 | Trichoptera | - | X |
| *Hydropsyche pellucidula* | CURTIS, 1834 | Trichoptera | X | X |
| *Hydropsyche saxonica* | McLACHLAN, 1884 | Trichoptera | - | X |
| *Hydropsyche siltalai* | DÖHLER, 1963 | Trichoptera | X | X |
| *Lepidostoma basale* | F. KOLENATI, 1848 | Trichoptera | - | X |
| *Lepidostoma hirtum* | FABRICIUS, 1775 | Trichoptera | X | X |
| *Limnephilus lunatus* | CURTIS, 1834 | Trichoptera | X | - |
| *Lype phaeopa* | J.F. STEPHENS, 1836 | Trichoptera | - | X |
| *Lype reducta* | HAGEN, 1868 | Trichoptera | X | X |
| *Micrasema longulum* | McLACHLAN, 1876 | Trichoptera | - | X |
| *Mystacides azurea* | LINNAEUS, 1761 | Trichoptera | - | X |
| *Mystacides nigra* | LINNAEUS, 1758 | Trichoptera | - | X |
| *Neureclipsis bimaculata* | LINNAEUS, 1758 | Trichoptera | - | X |
| *Odontocerum albicorne* | SCOPOLI, 1763 | Trichoptera | - | X |
| *Philopotamus montanus* | E. DONOVAN, 1813 | Trichoptera | - | X |
| *Plectrocnemia conspersa* | CURTIS, 1834 | Trichoptera | - | X |
| *Polycentropus flavomaculatus* | PICTET, 1834 | Trichoptera | - | X |
| *Polycentropus irroratus* | CURTIS, 1835 | Trichoptera | X | X |
| *Potamophylax cingulatus* | STEPHENS, 1837 | Trichoptera | X | X |
| *Potamophylax latipennis* | CURTIS, 1834 | Trichoptera | X | X |
| *Potamophylax luctuosus* | PILLER & MITTERPACHER, 1783 | Trichoptera | X | X |
| *Potamophylax rotundipennis* | BRAUER, 1857 | Trichoptera | - | X |
| *Psychomyia pusilla* | FABRICIUS, 1781 | Trichoptera | - | X |
| *Rhyacophila dorsalis* | CURTIS, 1834 | Trichoptera | - | X |
| *Rhyacophila evoluta* | McLACHLAN, 1879 | Trichoptera | - | X |
| *Rhyacophila fasciata* | HAGEN, 1859 | Trichoptera | - | X |
| *Rhyacophila nubila* | ZETTERSTEDT, 1840 | Trichoptera | - | X |
| *Rhyacophila obliterata* | McLACHLAN, 1863 | Trichoptera | - | X |
| *Rhyacophila tristis* | PICTET, 1834 | Trichoptera | - | X |
| *Sericostoma flavicorne* | SCHNEIDER, 1845 | Trichoptera | - | X |
| *Sericostoma personatum* | KIRBY & SPENCER, 1826 | Trichoptera | - | X |
| *Silo nigricornis* | PICTET, 1834 | Trichoptera | - | X |
| *Silo pallipes* | FABRICIUS, 1781 | Trichoptera | - | X |
| *Silo piceus* | BRAUER, 1857 | Trichoptera | - | X |
| *Dendrocoelum lacteum* | O.F. MÜLLER, 1774 | Turbellaria | - | X |
| *Dugesia gonocephala* | DUGES, 1830 | Turbellaria | X | X |
| *Dugesia lugubris* | SCHMIDT, 1861 | Turbellaria | - | X |
| *Dugesia polychroa* | SCHMIDT, 1861 | Turbellaria | - | X |
| *Polycelis felina* | DALYELL, 1814 | Turbellaria | - | X |
| *Polycelis nigra* | MUELLER, 1774 | Turbellaria | - | X |
| *Polycelis tenuis* | IJIMA, 1884 | Turbellaria | - | X |

**Table ST2** EURO-CORDEX models and versions used in this study (from Kiesel et al., 2019).

| **ID** | **GCM** | **GCMshort** | **RCM** | **RCMshort** |
| --- | --- | --- | --- | --- |
| 1 | CNRM-CERFACS-CNRM-CM5 | CNRM | CLMcom-CCLM4-8-17 | CLMcom |
| 2 | CNRM-CERFACS-CNRM-CM5 | CNRM | CNRM-ALADIN53 | ALADIN |
| 3 | CNRM-CERFACS-CNRM-CM5 | CNRM | SMHI-RCA4 | SMHI |
| 4 | ICHEC-EC-EARTH | ICHEC | CLMcom-CCLM4-8-17 | CLMcom |
| 5 | ICHEC-EC-EARTH | ICHEC | DMI-HIRHAM5 | DMI |
| 6 | ICHEC-EC-EARTH | ICHEC | KNMI-RACMO22E | KNMI |
| 7 | ICHEC-EC-EARTH | ICHEC | SMHI-RCA4 | SMHI |
| 8 | IPSL-IPSL-CM5A-MR | IPSL | IPSL-INERIS-WRF331F | INERIS |
| 9 | IPSL-IPSL-CM5A-MR | IPSL | SMHI-RCA4 | SMHI |
| 10 | MOHC-HadGEM2-ES | MOHC | CLMcom-CCLM4-8-17 | CLMcom |
| 11 | MOHC-HadGEM2-ES | MOHC | KNMI-RACMO22E | KNMI |
| 12 | MOHC-HadGEM2-ES | MOHC | SMHI-RCA4 | SMHI |
| 13 | MPI-M-MPI-ESM-LR | MPI | CLMcom-CCLM4-8-17 | CLMcom |
| 14 | MPI-M-MPI-ESM-LR | MPI | MPI-CSC-REMO2009v1 | REMO1 |
| 15 | MPI-M-MPI-ESM-LR | MPI | MPI-CSC-REMO2009v2 | REMO2 |
| 16 | MPI-M-MPI-ESM-LR | MPI | SMHI-RCA4 | SMHI |

**Table ST3** The Mean±SD of percent change of high flow conditions (dh4) for each of 16 GCMs and RCMs in northern lowlands (Treene catchment) and the central lower mountainous region (Kinzig catchment) in each horizon of 2050 and 2090. According to Fig. 3.

| Catchment | Climate model | horizon 2050 | | horizon 2090 | |
| --- | --- | --- | --- | --- | --- |
|  |  | Mean±SD | CV | Mean±SD | CV |
| Treene | 1 | -6.2±1.8 | -29.03 | -2.1±2.2 | -104.76 |
| Treene | 2 | 22.2±1.3 | 5.86 | 18.5±1.3 | 7.03 |
| Treene | 3 | 7.2±0.7 | 10 | 5.3±4.2 | 79.25 |
| Treene | 4 | 3.2±2 | 62.5 | 8.4±2.9 | 34.52 |
| Treene | 5 | -0.4±1.7 | -425 | 14.6±3.2 | 21.92 |
| Treene | 6 | 11.4±1.8 | 15.79 | 20.1±3.2 | 15.92 |
| Treene | 7 | 8.6±2.5 | 29.07 | 15.2±2.6 | 17.11 |
| Treene | 8 | -2.8±2.7 | -96.43 | 7.5±1.8 | 24 |
| Treene | 9 | -1.6±1.4 | -87.5 | 11.4±1.6 | 14.04 |
| Treene | 10 | 7.5±2.7 | 36 | 6.5±2.2 | 33.85 |
| Treene | 11 | 19.7±2.9 | 14.72 | 15.6±3.3 | 21.15 |
| Treene | 12 | -21.2±2.3 | -10.85 | 6.1±1 | 16.39 |
| Treene | 13 | 4±1.7 | 42.5 | 4.8±1.3 | 27.08 |
| Treene | 14 | 20.5±3.5 | 17.07 | 15.3±1.9 | 12.42 |
| Treene | 15 | 35.6±2.4 | 6.74 | 42.8±1.4 | 3.27 |
| Treene | 16 | 19.3±0.9 | 4.66 | 22.4±1.3 | 5.8 |
| Kinzig | 1 | -15.3±3.4 | -22.22 | -19.6±2.8 | -14.29 |
| Kinzig | 2 | -1.7±6.3 | -370.59 | -13±5.9 | -45.38 |
| Kinzig | 3 | -2.2±3.6 | -163.64 | -11.9±2.1 | -17.65 |
| Kinzig | 4 | 16.8±3.5 | 20.83 | 6.5±2.2 | 33.85 |
| Kinzig | 5 | -3.6±4.6 | -127.78 | -1.8±4 | -222.22 |
| Kinzig | 6 | -7.6±3.6 | -47.37 | 4.6±7.3 | 158.7 |
| Kinzig | 7 | 23.1±3.9 | 16.88 | 15±2.8 | 18.67 |
| Kinzig | 8 | 7.6±4.3 | 56.58 | 15.5±3.5 | 22.58 |
| Kinzig | 9 | -3.1±2.1 | -67.74 | 26.6±7.2 | 27.07 |
| Kinzig | 10 | -9.6±1.4 | -14.58 | -0.7±2.5 | -357.14 |
| Kinzig | 11 | 2.5±1.9 | 76 | -3.7±2.9 | -78.38 |
| Kinzig | 12 | -2.1±3.5 | -166.67 | 3.1±4.5 | 145.16 |
| Kinzig | 13 | -10.9±2 | -18.35 | 2.6±1.3 | 50 |
| Kinzig | 14 | -17.7±3.8 | -21.47 | -15.1±3.8 | -25.17 |
| Kinzig | 15 | 26.1±4 | 15.33 | 35±5.5 | 15.71 |
| Kinzig | 16 | 16.8±8.2 | 48.81 | 20.1±3.8 | 18.91 |

**Table ST4** The Mean±SD and coefficient of variation (CV) of relative changes in species’ abundance over 16 GCMs and RCMs in the northern lowlands (Treene catchment) in each horizon of 2050 and 2090. According to Fig. 4.

| **Catchment** | **Species** | **Horizon 2050** | | **Horizon 2050** | |
| --- | --- | --- | --- | --- | --- |
|  |  | **Mean±SD** | **CV** | **Mean±SD** | **CV** |
| Treene | Pisidium amnicum | -2.75±6.29 | -228.64 | -4.96±4.64 | -93.48 |
| Treene | Pisidium subtruncatum | -2.48±5.06 | -203.82 | -4.42±3.7 | -83.9 |
| Treene | Pisidium supinum | -2.2±4.43 | -201.14 | -3.91±3.27 | -83.51 |
| Treene | Sphaerium corneum | 1.83±3.52 | 192.4 | 3.14±2.44 | 77.74 |
| Treene | Elmis aenea | 4.35±7.59 | 174.57 | 7.31±5.46 | 74.65 |
| Treene | Elmis maugetii | 4.93±9.12 | 185.12 | 8.41±6.84 | 81.39 |
| Treene | Elmis rietscheli | 4.77±8.32 | 174.44 | 8.05±5.97 | 74.15 |
| Treene | Elmis rioloides | 5.03±8.81 | 175.04 | 8.48±6.4 | 75.4 |
| Treene | Elodes minuta | 1.36±2.56 | 188.54 | 1.71±3.37 | 196.65 |
| Treene | Limnius volckmari | 1.7±2.59 | 152.37 | 2.95±3.45 | 116.65 |
| Treene | Orectochilus villosus | 2.59±8.2 | 316.79 | 5.6±7.13 | 127.23 |
| Treene | Oulimnius tuberculatus | 7.83±12.93 | 165.17 | 13.07±10.6 | 81.04 |
| Treene | Asellus aquaticus | -0.17±0.3 | -172.37 | -0.29±0.24 | -80.21 |
| Treene | Gammarus pulex | 0.01±0.56 | 8876.82 | 0.07±0.52 | 706.58 |
| Treene | Proasellus coxalis | 2±3.58 | 178.59 | 3.34±2.7 | 80.88 |
| Treene | Atherix ibis | 3.11±5.03 | 161.9 | 5.2±4.41 | 84.7 |
| Treene | Prodiamesa olivacea | -1.37±4.65 | -340.25 | -3.42±3.09 | -90.18 |
| Treene | Ptychoptera paludosa | 0.24±1.97 | 823.47 | -0.46±1.77 | -385.85 |
| Treene | Simulium equinum | 5.42±8.87 | 163.53 | 8.66±6.67 | 77.05 |
| Treene | Simulium ornatum | 5.31±8.66 | 162.96 | 8.49±6.47 | 76.24 |
| Treene | Baetis atrebatinus | 3.97±6.26 | 157.94 | 6.52±5.59 | 85.66 |
| Treene | Baetis fuscatus | 3.97±6.26 | 157.94 | 6.52±5.59 | 85.66 |
| Treene | Baetis rhodani | -0.76±1.78 | -234.83 | -1.38±1.4 | -101.85 |
| Treene | Baetis vernus | 3.68±5.79 | 157.12 | 6.07±5.18 | 85.29 |
| Treene | Caenis horaria | 4.34±7.65 | 176.2 | 7.39±5.47 | 74.06 |
| Treene | Caenis rivulorum | 2.38±4.84 | 203.28 | 3.91±3.08 | 78.77 |
| Treene | Ephemera danica | 0.54±0.96 | 177.97 | 0.93±0.74 | 80.05 |
| Treene | Heptagenia sulphurea | 6.32±10.35 | 163.9 | 10.23±7.91 | 77.34 |
| Treene | Leptophlebia submarginata | 5.33±10.6 | 198.85 | 9.45±8.25 | 87.31 |
| Treene | Ancylus fluviatilis | 3.59±5.46 | 152.32 | 6.38±5.44 | 85.22 |
| Treene | Anisus vortex | -2.08±3.93 | -189.13 | -3.07±3.53 | -115.3 |
| Treene | Bithynia leachii | -1.28±2.13 | -166.29 | -1.99±2.03 | -102.26 |
| Treene | Bithynia tentaculata | -0.66±1.19 | -179.99 | -1.14±0.92 | -80.56 |
| Treene | Planorbarius corneus | -1.89±3.99 | -211.05 | -3.53±2.77 | -78.39 |
| Treene | Planorbis planorbis | -1.92±3.82 | -198.56 | -3.35±2.72 | -81.4 |
| Treene | Radix balthica | -0.7±1.26 | -179.93 | -1.19±1.02 | -85.81 |
| Treene | Erpobdella nigricollis | -0.67±1.52 | -225.87 | -1.22±1.31 | -107.95 |
| Treene | Erpobdella octoculata | -1.11±2.06 | -184.39 | -1.94±1.59 | -81.59 |
| Treene | Glossiphonia complanata | -0.81±1.46 | -179.63 | -1.39±1.11 | -80.32 |
| Treene | Glossiphonia nebulosa | -1.59±3.26 | -205.03 | -2.92±2.61 | -89.23 |
| Treene | Sialis lutaria | -3.18±7.25 | -228.12 | -5.8±5.02 | -86.43 |
| Treene | Calopteryx splendens | 4.76±9.61 | 201.84 | 8.35±7.16 | 85.81 |
| Treene | Lumbriculus variegatus | -1.29±2.63 | -204.49 | -2.27±1.84 | -81.04 |
| Treene | Isoperla grammatica | 4.5±9.62 | 213.99 | 8.08±7.22 | 89.33 |
| Treene | Nemoura cinerea | -1.82±3.2 | -175.89 | -2.93±2.61 | -88.95 |
| Treene | Anabolia nervosa | 1.32±5.81 | 439.88 | 2.94±4.87 | 165.67 |
| Treene | Athripsodes albifrons | 1.38±5.6 | 405.7 | 1.95±4.91 | 251.67 |
| Treene | Athripsodes cinereus | 1.38±5.6 | 405.7 | 1.95±4.91 | 251.67 |
| Treene | Halesus radiatus | -3.38±5.7 | -168.6 | -5.52±5.09 | -92.17 |
| Treene | Hydropsyche angustipennis | 4.39±8.9 | 202.75 | 7.53±6.35 | 84.3 |
| Treene | Hydropsyche pellucidula | 2.86±7.96 | 278.76 | 5.58±6.53 | 117.02 |
| Treene | Hydropsyche siltalai | 0.47±0.84 | 179.25 | 0.81±0.63 | 78.32 |
| Treene | Lepidostoma hirtum | 48.86±71.5 | 146.32 | 64.86±55.54 | 85.64 |
| Treene | Limnephilus lunatus | -0.75±1.29 | -173.23 | -1.28±1.06 | -82.4 |
| Treene | Lype reducta | 1.5±2.55 | 170.01 | 2.6±2.15 | 82.63 |
| Treene | Polycentropus irroratus | 5.66±9.88 | 174.72 | 9.85±7.72 | 78.37 |
| Treene | Potamophylax cingulatus | 2.39±7.28 | 304.48 | 4.79±5.75 | 120.1 |
| Treene | Potamophylax latipennis | 2.39±7.28 | 304.48 | 4.79±5.75 | 120.1 |
| Treene | Potamophylax luctuosus | 2.77±8.12 | 293.71 | 5.43±6.2 | 114.06 |
| Treene | Dugesia gonocephala | 4.88±7.93 | 162.25 | 7.86±6.75 | 85.85 |

**Table ST5** The Mean±SD and coefficient of variation (CV) of relative changes in species’ abundance over 16 GCMs and RCMs in the central lower mountainous region (Kinzig catchment) in each horizon of 2050 and 2090. According to Fig. 4.

| **Catchment** | **Species** | **Horizon 2050** | | **Horizon 2090** | |
| --- | --- | --- | --- | --- | --- |
|  |  | **Mean±SD** | **CV** | **Mean±SD** | **CV** |
| Kinzig | Pisidium amnicum | 9.19±21.51 | 234.19 | 2.58±18.27 | 707.49 |
| Kinzig | Pisidium casertanum | 0.16±3.14 | 2020.41 | -0.02±3.62 | -15780.12 |
| Kinzig | Pisidium subtruncatum | 0.19±2.01 | 1071.93 | -0.34±2.19 | -643.54 |
| Kinzig | Sphaerium corneum | 9.19±18.37 | 199.89 | 5.41±12.98 | 240.09 |
| Kinzig | Elmis aenea | 0.57±6.5 | 1132.65 | 2.5±10.97 | 438.21 |
| Kinzig | Elmis maugetii | 0.59±5.75 | 975.38 | 5.07±13.97 | 275.44 |
| Kinzig | Elmis rietscheli | 0.23±3.68 | 1585.05 | -0.06±4.65 | -7677.77 |
| Kinzig | Elmis rioloides | 0.23±3.68 | 1585.05 | -0.06±4.65 | -7677.77 |
| Kinzig | Hydraena dentipes | 1.07±16.25 | 1512.35 | 0.8±23.97 | 2996.9 |
| Kinzig | Hydraena gracilis | 1.11±6.21 | 561.77 | 0.82±8.57 | 1046.32 |
| Kinzig | Hydraena minutissima | 0.05±6.44 | 12427.98 | 0.12±9.74 | 8130.03 |
| Kinzig | Limnius perrisi | 0.22±7.94 | 3616.36 | 1.17±12.41 | 1057.5 |
| Kinzig | Limnius volckmari | -0.12±1.41 | -1184.6 | -0.44±1.53 | -346.77 |
| Kinzig | Orectochilus villosus | 0.12±5.07 | 4347.05 | 1.38±9.03 | 653.82 |
| Kinzig | Oulimnius tuberculatus | -0.07±4.22 | -5836.07 | 2.66±9.8 | 368.87 |
| Kinzig | Platambus maculatus | -2.12±13.56 | -640.57 | -1.82±17.99 | -987.1 |
| Kinzig | Asellus aquaticus | 3.8±7.69 | 202.55 | 2.94±5.56 | 189.15 |
| Kinzig | Gammarus fossarum | 0.31±4.3 | 1389.31 | 0.31±6.1 | 1956.22 |
| Kinzig | Gammarus pulex | 4.18±14.89 | 356.04 | 8.44±15.65 | 185.41 |
| Kinzig | Gammarus roeselii | 1.2±12.7 | 1062.03 | 6.85±12.98 | 189.65 |
| Kinzig | Atherix ibis | -0.13±4.94 | -3895.1 | 5.26±13.1 | 249.02 |
| Kinzig | Atrichops crassipes | 42.01±44.22 | 105.28 | 30.53±57.79 | 189.26 |
| Kinzig | Chironomus riparius | 166.38±354.08 | 212.81 | 142.88±263.35 | 184.32 |
| Kinzig | Prodiamesa olivacea | 0.08±1.81 | 2253.12 | -0.92±2.72 | -297.56 |
| Kinzig | Simulium argyreatum | 2.19±5.14 | 235.01 | 0.49±4.41 | 905.07 |
| Kinzig | Simulium cryophilum | 0.12±2.67 | 2227.06 | -0.26±3.35 | -1265.71 |
| Kinzig | Simulium ornatum | 17.25±53.91 | 312.44 | 29.24±63.82 | 218.24 |
| Kinzig | Simulium variegatum | 4.13±9.88 | 239.46 | 3.11±9.93 | 319.47 |
| Kinzig | Simulium vernum | 16.19±28.4 | 175.43 | 14.37±31.18 | 216.95 |
| Kinzig | Baetis alpinus | 3.73±13.8 | 369.82 | 4.64±12.32 | 265.28 |
| Kinzig | Baetis fuscatus | 0.27±8.4 | 3057.21 | 0.06±11.6 | 20490.83 |
| Kinzig | Baetis lutheri | 37.61±83.92 | 223.13 | 81.13±161.97 | 199.64 |
| Kinzig | Baetis muticus | 0.57±1.87 | 329.88 | 0.17±2.14 | 1221.91 |
| Kinzig | Baetis niger | 0.35±1.52 | 439.7 | 0.25±2.15 | 842.74 |
| Kinzig | Baetis rhodani | 1.18±6.67 | 566.86 | 2.89±5.07 | 175.72 |
| Kinzig | Baetis vernus | 1.69±17.79 | 1054.9 | 6.95±18.81 | 270.6 |
| Kinzig | Centroptilum luteolum | -0.13±5.02 | -3881.55 | -1.15±6.47 | -563.98 |
| Kinzig | Ecdyonurus dispar | -0.97±3.71 | -384.13 | -1.62±4.73 | -291.65 |
| Kinzig | Ecdyonurus macani | 0.05±2.16 | 4047.91 | -0.94±3.27 | -347.75 |
| Kinzig | Ecdyonurus submontanus | 0.35±4.68 | 1321.87 | -0.21±6.06 | -2950.77 |
| Kinzig | Ecdyonurus torrentis | 0.81±1.59 | 195.58 | 1.16±2.1 | 181.54 |
| Kinzig | Ecdyonurus venosus | 0.02±4.27 | 23196.97 | -0.32±5.76 | -1792.69 |
| Kinzig | Electrogena affinis | 11.6±46.8 | 403.32 | 20.5±95.9 | 467.89 |
| Kinzig | Epeorus assimilis | 0.33±2.33 | 707.91 | 1.83±4.74 | 259.24 |
| Kinzig | Ephemera danica | -0.05±4.69 | -9568.52 | 1.64±4.49 | 273.26 |
| Kinzig | Ephemerella mucronata | 0.7±21.6 | 3084.16 | 15.99±59.5 | 372.13 |
| Kinzig | Habroleptoides confusa | 0.05±1.95 | 4035.14 | -0.14±3.05 | -2150.73 |
| Kinzig | Habrophlebia lauta | 0.37±0.53 | 143.67 | 0.53±1.49 | 279.95 |
| Kinzig | Heptagenia sulphurea | 0.09±6.87 | 7711.36 | -1.14±11.02 | -963.72 |
| Kinzig | Paraleptophlebia submarginata | 0.22±3.59 | 1666.87 | -0.09±4.7 | -5279.64 |
| Kinzig | Rhithrogena semicolorata | 0.2±1.13 | 557.51 | 0.79±2.14 | 271.25 |
| Kinzig | Serratella ignita | 0.48±2.54 | 527.82 | 0.52±3.35 | 642.63 |
| Kinzig | Torleya major | 7.88±17.06 | 216.42 | 24.17±54.19 | 224.16 |
| Kinzig | Ancylus fluviatilis | 1.13±8.01 | 709.02 | 1.18±10.23 | 870.41 |
| Kinzig | Bithynia tentaculata | 11.44±39.19 | 342.49 | 7.83±56.34 | 719.31 |
| Kinzig | Potamopyrgus antipodarum | 67.67±131.1 | 193.73 | 47.05±96 | 204.04 |
| Kinzig | Radix balthica | -0.21±6.08 | -2891.81 | -1.47±6.88 | -468.89 |
| Kinzig | Radix labiata | 2.69±9.91 | 368.27 | 5.49±12.89 | 234.67 |
| Kinzig | Aphelocheirus aestivalis | 3.29±33.44 | 1016.28 | 13.23±32.48 | 245.52 |
| Kinzig | Erpobdella nigricollis | -0.24±4.04 | -1655.21 | 0.77±4.44 | 574.75 |
| Kinzig | Erpobdella octoculata | 1.62±6.42 | 395.3 | -0.28±6.06 | -2126.48 |
| Kinzig | Erpobdella vilnensis | 0.02±1.66 | 6970.38 | 1.66±4.19 | 251.65 |
| Kinzig | Glossiphonia complanata | -0.68±4.25 | -628.88 | -0.32±6.34 | -1973.81 |
| Kinzig | Glossiphonia nebulosa | 0.13±7.42 | 5721.78 | -0.8±9.7 | -1213.5 |
| Kinzig | Helobdella stagnalis | 0.31±6.19 | 2028.63 | -0.08±4.6 | -5962.32 |
| Kinzig | Sialis fuliginosa | 8.31±23.31 | 280.38 | 9.26±22.75 | 245.62 |
| Kinzig | Sialis lutaria | 0.47±6.32 | 1354.15 | -3.54±8.15 | -230.25 |
| Kinzig | Calopteryx splendens | -1.48±4.99 | -338 | -2.51±4.99 | -199 |
| Kinzig | Calopteryx virgo | -0.08±8.13 | -10355.35 | 0.93±13.3 | 1426.43 |
| Kinzig | Eiseniella tetraedra | 1.62±5.13 | 317.28 | 0.35±4.55 | 1302.21 |
| Kinzig | Lumbriculus variegatus | 6.01±14.26 | 237.35 | 6.44±19.2 | 298.29 |
| Kinzig | Stylodrilus heringianus | 6.29±15.48 | 246.02 | 10.88±16.91 | 155.38 |
| Kinzig | Brachyptera risi | 1.41±2.9 | 205.42 | 0.92±2.38 | 259.97 |
| Kinzig | Brachyptera seticornis | 0.62±2.27 | 367.35 | 0.09±2.92 | 3107.69 |
| Kinzig | Leuctra hippopus | -0.68±13.51 | -1982.66 | -1.88±17.83 | -950.08 |
| Kinzig | Leuctra nigra | 0.06±4.71 | 7817.13 | -0.23±6.35 | -2777.93 |
| Kinzig | Siphonoperla torrentium | 0.18±1.12 | 609.29 | -0.09±1.11 | -1308.38 |
| Kinzig | Spongilla lacustris | 13.9±30.43 | 218.9 | 12.58±21 | 166.95 |
| Kinzig | Anabolia nervosa | 3.45±7.22 | 209.12 | 4.39±8.13 | 185.14 |
| Kinzig | Anomalopterygella chauviniana | 0.1±0.87 | 878.05 | 0.06±1.49 | 2314.41 |
| Kinzig | Athripsodes albifrons | 31.3±55.64 | 177.75 | 35.32±96.33 | 272.73 |
| Kinzig | Athripsodes bilineatus | 21.67±62.3 | 287.48 | 7.82±49.18 | 629.07 |
| Kinzig | Athripsodes cinereus | 4.82±14.93 | 309.73 | 2.56±18.88 | 736.93 |
| Kinzig | Brachycentrus subnubilus | 6.18±39.65 | 641.99 | 16.9±37.71 | 223.19 |
| Kinzig | Ceraclea albimacula | 11.34±49.72 | 438.38 | 23.82±66.71 | 280.11 |
| Kinzig | Ceraclea dissimilis | 12.18±21.72 | 178.41 | 7.04±22.8 | 324.04 |
| Kinzig | Chaetopteryx villosa | 2.16±3.56 | 165.16 | 3.78±5.8 | 153.41 |
| Kinzig | Cyrnus trimaculatus | 4.87±34.16 | 701.92 | 17.61±36.77 | 208.79 |
| Kinzig | Drusus annulatus | 0.11±3.51 | 3292.54 | -0.62±3.54 | -575.24 |
| Kinzig | Goera pilosa | 0.14±4.42 | 3253.34 | -1.3±5.27 | -404.47 |
| Kinzig | Halesus digitatus | 0.9±5.58 | 622.92 | -1.32±7.07 | -536.41 |
| Kinzig | Halesus radiatus | 0.58±5.77 | 1002.63 | 0.29±8.14 | 2814.73 |
| Kinzig | Hydropsyche angustipennis | 5.53±11.73 | 211.89 | 11.87±17.13 | 144.32 |
| Kinzig | Hydropsyche incognita | 0.04±4.02 | 8949.09 | -0.72±7.06 | -984.54 |
| Kinzig | Hydropsyche instabilis | 0.63±8.95 | 1413.44 | 2.12±14.57 | 686.07 |
| Kinzig | Hydropsyche pellucidula | 2.88±9.36 | 324.61 | 2.02±12.57 | 623.85 |
| Kinzig | Hydropsyche saxonica | 0.45±10.84 | 2411.57 | 0.38±14.87 | 3883.3 |
| Kinzig | Hydropsyche siltalai | 0.48±1.19 | 247.21 | 0.42±1.3 | 308.18 |
| Kinzig | Lepidostoma basale | -0.04±3.72 | -9822.36 | 0.63±4.47 | 710.02 |
| Kinzig | Lepidostoma hirtum | 1.56±12.2 | 783.75 | 0.64±14.95 | 2342.24 |
| Kinzig | Lype phaeopa | 51.83±125.99 | 243.07 | 69.26±131.47 | 189.81 |
| Kinzig | Lype reducta | 0.07±3.79 | 5523.64 | -1.23±4.94 | -400.42 |
| Kinzig | Micrasema longulum | -0.26±4.46 | -1727.84 | 0.4±5.82 | 1466.29 |
| Kinzig | Mystacides azurea | 0.21±4.41 | 2128.5 | -1.05±6.66 | -633.23 |
| Kinzig | Mystacides nigra | -3.08±11.08 | -359.63 | -1.54±11.04 | -718.72 |
| Kinzig | Neureclipsis bimaculata | 6.78±24.92 | 367.8 | 10.38±26.12 | 251.71 |
| Kinzig | Odontocerum albicorne | 0.38±3.83 | 1019.78 | 0.04±5.16 | 13650.11 |
| Kinzig | Philopotamus montanus | 3.17±10.11 | 318.51 | 4.81±13.44 | 279.42 |
| Kinzig | Plectrocnemia conspersa | 0.16±0.8 | 495.41 | 0.01±0.78 | 6816.92 |
| Kinzig | Polycentropus flavomaculatus | 4.37±9.11 | 208.29 | 13.7±15.63 | 114.11 |
| Kinzig | Polycentropus irroratus | 43.09±61.22 | 142.09 | 96.97±122.07 | 125.89 |
| Kinzig | Potamophylax cingulatus | -0.13±2.76 | -2047.11 | -0.4±3.13 | -773.49 |
| Kinzig | Potamophylax latipennis | 0±2.35 | -99473.3 | -0.65±2.9 | -446.39 |
| Kinzig | Potamophylax luctuosus | 0.02±2.46 | 10492.68 | -0.5±2.79 | -558.72 |
| Kinzig | Potamophylax rotundipennis | 3.93±29.56 | 751.92 | 3.09±34.64 | 1122.34 |
| Kinzig | Psychomyia pusilla | 0.25±2.68 | 1059.9 | 0.24±3.77 | 1573.85 |
| Kinzig | Rhyacophila dorsalis | -0.3±2.96 | -973.23 | 2.04±8.71 | 426.93 |
| Kinzig | Rhyacophila evoluta | 0.09±1.4 | 1519.34 | -0.32±1.44 | -451.4 |
| Kinzig | Rhyacophila fasciata | 0.69±11.52 | 1660.16 | 4.05±17.53 | 432.55 |
| Kinzig | Rhyacophila nubila | 22.11±46.95 | 212.3 | 25.5±56.8 | 222.79 |
| Kinzig | Rhyacophila obliterata | -0.41±2.83 | -688.96 | 0.36±4.79 | 1339.39 |
| Kinzig | Rhyacophila tristis | -3.3±6.28 | -190.06 | -3.16±6.61 | -209.52 |
| Kinzig | Sericostoma flavicorne | -0.13±2.35 | -1819.93 | 0.28±2.81 | 1015.33 |
| Kinzig | Sericostoma personatum | 0.07±1.04 | 1571.93 | -0.19±1.07 | -566.4 |
| Kinzig | Silo nigricornis | 0.37±1.26 | 343.36 | 0.45±1.78 | 396.26 |
| Kinzig | Silo pallipes | 0.31±2.15 | 692.33 | 0.09±2.73 | 3056.12 |
| Kinzig | Silo piceus | 1.41±4.61 | 325.78 | 0.78±5.69 | 724.63 |
| Kinzig | Dendrocoelum lacteum | 4.18±8.49 | 202.76 | 4.25±7.6 | 178.71 |
| Kinzig | Dugesia gonocephala | 0.87±5.94 | 685.85 | 1.35±7.07 | 525.23 |
| Kinzig | Dugesia lugubris | -0.13±5.25 | -3977.83 | -2.34±7.6 | -324.63 |
| Kinzig | Dugesia polychroa | 1.08±6.18 | 572.06 | -2.39±6.33 | -264.72 |
| Kinzig | Polycelis felina | 3.66±17.14 | 467.88 | -0.34±16.08 | -4765.97 |
| Kinzig | Polycelis nigra | 36.36±83.86 | 230.63 | 59.79±88.92 | 148.73 |
| Kinzig | Polycelis tenuis | -2.49±15.39 | -617.76 | -7.13±16.36 | -229.58 |
